# DOPA decarboxylase is an emerging biomarker for Parkinsonian disorders including preclinical Lewy body disease

**DOI:** 10.1038/s43587-023-00478-y

**Published:** 2023-09-18

**Authors:** Joana B. Pereira, Atul Kumar, Sara Hall, Sebastian Palmqvist, Erik Stomrud, Divya Bali, Piero Parchi, Niklas Mattsson-Carlgren, Shorena Janelidze, Oskar Hansson

**Affiliations:** 1 Division of Neuro, Department of Clinical Neutaroscience, Karolinska Institutet, Solna, Sweden; 2 Clinical Memory Research Unit, Department of Clinical Sciences, Lund University, Malmö, Sweden; 3 Memory Clinic, Skåne University Hospital, Malmö, Sweden; 4 Istituto di Ricovero e Cura a Carattere Scientifico, Istituto delle Scienze Neurologiche di Bologna, Bologna, Italy; 5 Department of Biomedical and Neuromotor Sciences, University of Bologna, Bologna, Italy; 6 Department of Neurology, Skåne University Hospital, Lund University, Lund, Sweden; 7 Wallenberg Center for Molecular Medicine, Lund University, Lund, Sweden

**Keywords:** Diagnostic markers, Movement disorders, Ageing

## Abstract

The diagnosis of Parkinsonian disorders is currently based on clinical criteria, which have limited sensitivity until most dopaminergic neurons are lost. Here we show that cerebrospinal fluid levels of DOPA decarboxylase (DDC) (also known as aromatic l-amino acid decarboxylase) can accurately identify patients with Lewy body disease (LBD) (area under the curve (AUC) = 0.89; *P*_FDR_ = 2.6 × 10^−13^) and are associated with worse cognitive performance (*P* < 0.05). We also found that DDC can detect preclinical LBD stages in clinically unimpaired individuals with a positive seed amplification α-synuclein assay (AUC = 0.81, *P* = 1.0 × 10^−5^) and that this biomarker could predict progression to clinical LBD over a 3-year period in preclinical cases (hazard ratio = 3.7 per s.d. change, confidence interval = 1.1–12.7). Moreover, DDC levels were also increased in atypical Parkinsonian disorders but not in non-Parkinsonian neurodegenerative disorders. These cerebrospinal fluid results were replicated in an independent cohort, where we also found that DDC levels in plasma could identify both LBD and atypical Parkinsonian disorders (AUC = 0.92, *P* = 1.3 × 10^−14^). Our results show that DDC might have a future role in clinical practice as a biomarker of dopaminergic dysfunction to detect Parkinsonian disorders even during the preclinical disease stages and predict their progression to clinical LBD.

## Abstract (web summary)

Pereira, Kumar et al. identify cerebrospinal fluid DOPA decarboxylase as a promising biomarker for Parkinsonian disorders that may be used for early preclinical detection of Parkinsonian disorders as well as determining the risk of conversion to Lewy body disease.

## Main

The burden of neurodegeneration on patients, caregivers and society is rapidly increasing together with greater life expectancy^1^. Parkinsonian disorders, including Lewy body disease (LBD) (Parkinson’s disease (PD), dementia with Lewy bodies (DLB)) and atypical Parkinsonian syndromes (PS) (multiple system atrophy (MSA), progressive supranuclear palsy (PSP), corticobasal syndrome (CBS)), are among the most common neurodegenerative disorders, affecting 6% of individuals worldwide and costing society more than 100 billion euros yearly^1^. Although their diagnosis is primarily based on clinical criteria, there is growing evidence indicating that the neurodegenerative processes underlying these disorders begin several years before the onset of clinical symptoms^2,3^. In addition, even when clinical criteria are correctly applied, the frequency of misdiagnosis is high due to considerable symptom overlap with other disorders^4^. Therefore, there is an urgent need to improve the diagnosis of Parkinsonian disorders, particularly in the early disease stages, to apply disease-modifying therapies that prevent neurodegeneration.

Because molecular changes in the brain are reflected in the cerebrospinal fluid (CSF), the CSF represents a valuable source of biomarkers for the early diagnosis of neurodegenerative disorders^5^. A successful example is Alzheimer’s disease (AD), where changes in CSF amyloid-β_1–42_ and phosphorylated tau isoforms reliably detect the underlying AD pathology even in the preclinical disease stages before the onset of overt clinical symptoms^6^. Similar efforts have been made in the search for CSF biomarkers for Parkinsonian disorders. For instance, due to the central role of α-synuclein misfolding in the development of LBD, several studies have assessed total α-synuclein in the CSF of patients with these disorders^7^. However, CSF total α-synuclein concentrations in LBD substantially overlap with those of controls, which limits their clinical use^8^. A remarkable breakthrough is the recent development of seed amplification assays (SAAs) for the CSF, which detect misfolded α-synuclein prone to aggregation and show high diagnostic accuracy in distinguishing LBD from controls, as well as the ability to detect Lewy body pathology^9–11^. However, most of these assays are not so useful in detecting atypical PS^4^.

To our knowledge, few studies have investigated potential biomarkers for all Parkinsonian disorders using a data-driven, large, multiplex proteomic approach. This type of methodology has the potential to identify pathbreaking disease biomarkers for early or more accurate diagnosis. Thus, to address this need, we measured an extensive panel of 2,943 proteins in the CSF and 92 proteins in plasma using a validated, highly sensitive and specific multiplex immunoassay developed by Olink Proteomics^12^. Our primary aim was to identify unique biomarkers that can detect clinical LBD and atypical Parkinsonian disorders. Our secondary aim was to find biomarkers that could detect preclinical LBD, which we defined as clinically unimpaired individuals (CUIs) with a positive SAA, reflecting underlying abnormal α-synuclein aggregation. Within this subsample of preclinical cases, we also aimed to use these unique biomarkers to predict their progression to clinical LBD over a 3-year follow-up. Our tertiary aim was to determine the specificity of the freshly identified biomarkers for Parkinsonian disorders versus other non-Parkinsonian neurodegenerative disorders, including AD, frontotemporal dementia (FTD) and vascular dementia. Finally, we replicated our findings in an independent sample to assess the generalizability of our results, and measured key candidate biomarkers in the blood (plasma).

## Results

**Fig. 1:**
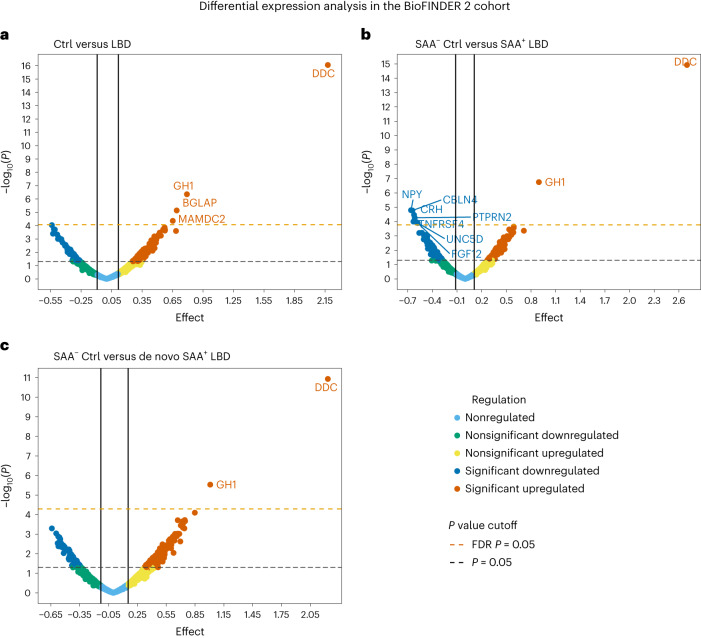
Differential expression analyses of patients with LBD compared to controls in BioFINDER 2. **a**, Results comparing all CUIs or controls (ctrl) to patients with LBD. **b**, Results comparing α-synuclein SAA^−^ controls to SAA^+^ patients with LBD. **c**, Results comparing α-synuclein SAA^−^ controls to SAA^+^ patients with de novo LBD. The upper horizontal indicates proteins that survived FDR correction. The strongest top hit corresponded to DDC (also known as aromatic l-amino acid decarboxylase).

**Fig. 2:**
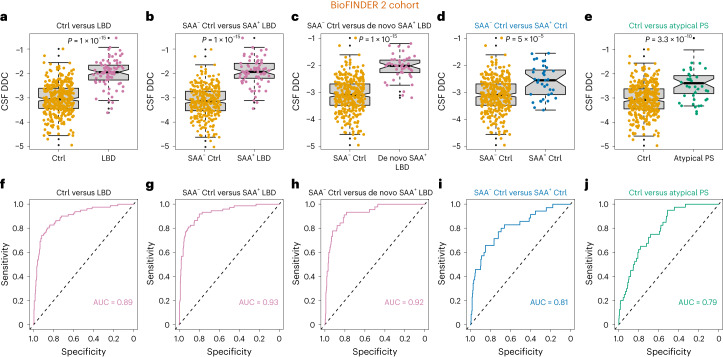
Increased CSF DDC levels in clinical LBD, preclinical LBD and atypical PS. **a**–**e**, Higher CSF levels were observed in (**a**) all patients with LBD, *n* = 428 independent samples (347 CUIs and 81 patients with LBD, including PD (*n* = 48) and DLB (*n* = 33)) (**b**), LBD with α-synuclein SAA^+^, *n* = 384 independent samples (310 CUIs and 74 patients with LBD) (**c**), de novo SAA^+^ patients with LBD, *n* = 355 independent samples (310 CUIs and 45 patients with LBD) (**d**), preclinical LBD defined as SAA^+^ CUIs or controls (SAA^+^ controls), *n* = 345 (SAA^+^ (*n* = 35); SAA^−^ (*n* = 310)) (**e**) and atypical PS compared to CUIs or controls, *n* = 387 independent samples (347 CUIs and 40 patients with PS). The boxes in **a**–**e** denote the limits of the interquartile range (IQR), which is calculated by dividing the median by the range of the data; the whiskers extend beyond the box to a maximum of 1.5 times the IQR. **f**–**j**, Results from the ROC analyses for the group comparisons, with the corresponding AUCs. (**f**), all patients with LBD, *n* = 428 independent samples (347 CUIs and 81 patients with LBD, including PD (*n* = 48) and DLB (*n* = 33)) (**g**), LBD with α-synuclein SAA+, *n* = 384 independent samples (310 CUIs and 74 patients with LBD) (**h**), de novo SAA+ patients with LBD, *n* = 355 independent samples (310 CUIs and 45 patients with LBD) (**i**), preclinical LBD defined as SAA+ CUIs or controls (SAA+ controls), *n* = 345 (SAA+ (*n* = 35); SAA− (*n* = 310)) (**j**) and atypical PS compared to CUIs or controls, *n* = 387 independent samples (347 CUIs and 40 patients with PS). All results were obtained using an analysis of covariance (ANCOVA) while adjusting for age and sex and controlling for multiple comparisons using Bonferroni correction. All *P* values are two-sided.

**Extended Data Fig. 1:**
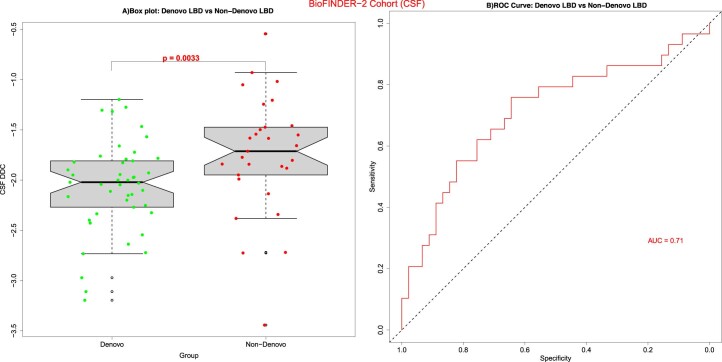
Increased CSF DDC levels in medicated SAA+ De novo LBD. Higher CSF DDC levels are observed in (**a**) medicated Lewy body disease (LBD) patients compared to drug naive LBD, n=74 [*de novo* SAA+ LBD patients (n=29), the medicated SAA+ LBD group (n=45)]. The box denotes the limits of the interquartile range, which is calculated by dividing the median by the range of the data, and the whiskers extend beyond the box to a maximum of 1.5 times the interquartile range. Results from the receiver operating curve analyses for the above group comparisons (**b**) with the corresponding areas under the curve (AUC). All results were obtained using an analysis of covariance (ANCOVA) while adjusting for age and sex and controlling for multiple comparisons using Bonferroni. All P values are two-sided.

To pursue these aims, we determined the CSF concentrations of the 2,943 proteins using the multiplex assay from Olink Proteomics in 347 CUIs (controls) and 81 patients with LBD, including PD (*n* = 48) and DLB (*n* = 33) (Supplementary Table 1) from the Swedish BioFINDER 2 cohort. We then conducted differential expression analysis on CSF protein levels with a generalized linear model while adjusting for age and sex and controlling for multiple comparisons using false discovery rate (FDR)^13^. When we compared patients with LBD to controls, ten proteins were significantly upregulated and four were downregulated in LBD (Supplementary Table 2). Among the upregulated proteins in LBD, a top hit with a stronger effect compared to the others (*β* = 2.2, *P*_FDR_ = 2.6 × 10^−13^; area under the curve (AUC) = 0.89, sensitivity = 0.83, specificity = 0.83) (Figs. 1a and 2a,f) was DOPA decarboxylase (DDC), also known as aromatic l-amino acid decarboxylase. DDC is an enzyme that converts levodopa into dopamine^14^, the latter being severely depleted in LBD due to the loss of dopaminergic neurons in the substantia nigra^15^. Similar results were found for DDC when the differential expression analysis was restricted to controls without underlying α-synuclein aggregation on an SAA (SAA^−^) (*n* = 310) and patients with LBD with α-synuclein aggregation on SAA (SAA^+^) (*n* = 74) (*β* = 2.7, *P*_FDR_ = 3.5 × 10^−12^; AUC = 0.93, sensitivity = 0.87, specificity = 0.86) (Figs. 1b and 2b,g) (Supplementary Table 3). Given that the most commonly used medication for the treatment of LBD includes a combination of levodopa and decarboxylase inhibitors, we repeated the analyses in patients with SAA^+^ LBD (*n* = 45) with early clinical disease who were not yet receiving any dopaminergic medications (that is, patients with de novo LBD) to assess their potential influence on our results. These analyses confirmed that DDC was the strongest top hit, being upregulated in patients with de novo LBD that were SAA^+^ compared to controls (*β* = 2.2, *P*_FDR_ = 3.4 × 10^−8^; AUC = 0.92, sensitivity = 0.84, specificity = 0.85) (Figs. 1c and 2c,h) (Supplementary Table 4). Within the group with LBD, no differences were found between patients with PD and DLB (*F*_2,79_ = 1.3, *p* = 0.09), but there were notable differences between controls and each of the previous subgroups (controls versus PD: *F*_2,391_ = 37.7, *P*_Bonferroni-corrected_ < 2 × 10^−15^; controls versus DLB: *F*_2,376_ = 43.4, *P*_Bonferroni-corrected_ < 2 × 10^−15^). Compared to patients with de novo SAA^+^ LBD (*n* = 29), the medicated group with SAA ^+^ LBD (*n* = 45) showed higher DDC levels (*F*_1,72_ = 6.7, *P* = 3.3 × 10^−3^, AUC = 0.71, sensitivity = 0.87, specificity = 0.45) (Extended Data Fig. 1a,b), in line with a recent study showing that levels of DDC enzyme activity are increased in PD and patients with atypical Parkinsonian symptoms who were on levodopa compared to de novo patients^14^.

Encouraged by these interesting results, we proceeded to investigate whether DDC could also be useful to identify the early preclinical (presymptomatic) stages of LBD by comparing CUIs (controls) who were SAA^+^ (*n* = 35) to those that were SAA^−^ (*n* = 310). These analyses showed that DDC was already significantly increased in these early disease stages compared to SAA^−^ controls (*F*_1,343_ = 20.1, *P*_Bonferroni-corrected_ = 5.0 × 10^−5^, AUC = 0.81, sensitivity = 0.77, specificity = 0.85) (Fig. 2d,i), highlighting its important clinical value for detecting LBD not only in clinically diagnosed LBD but also in individuals without any clinical impairment, making it a potential candidate marker for detection of preclinical LBD with relevance for early interventional trials. We did not find any significant differences in motor scores (Unified Parkinson’s Disease Rating Scale, part III (UPDRS III)) in SAA^+^ compared to SAA^−^ CUIs, but SAA^+^ individuals performed worse on two cognitive tests assessing global cognition (the modified Preclinical Alzheimer Cognitive Composite (mPACC) test: *P* = 0.017) and cognitive speed (A Quick Test of Cognitive Speed (AQT): *P* = 0.032).

**Extended Data Fig. 2:**
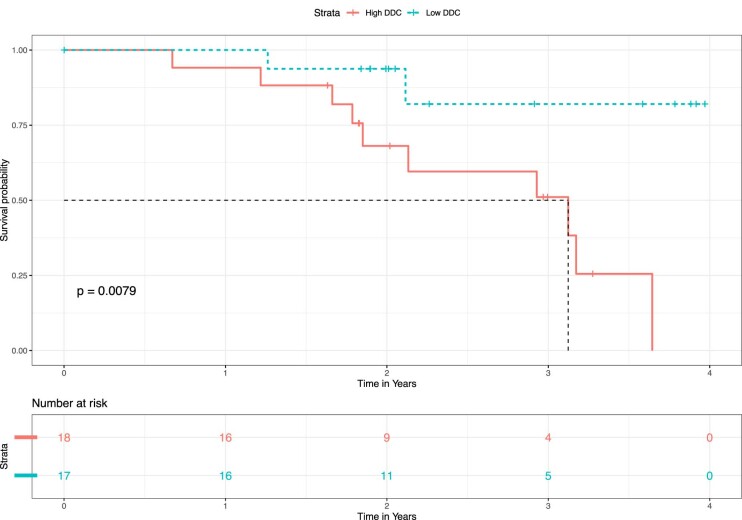
The Kaplan–Meier survival curve. The Kaplan–Meier survival curve of different DDC levels for clinically unimpaired SAA+ individuals. Samples were categorized into low-and high-DDC groups using a median NPX value of DDC.

In an additional analysis, we also tested if DDC could predict the progression to clinical LBD in SAA^+^, clinically normal cases (*n* = 35) who were assessed over a period of 2.53 ± 1.20 years (with clinical assessments every 1–2 years). Out of 35, 12 individuals progressed to clinical LBD. None of the SAA^−^, clinically normal cases progressed to clinical LBD during follow-up. Higher DDC levels were significantly associated with subsequent progression to clinical LBD (hazard ratio (HR) = 3.7 per s.d. change, confidence interval (CI) = 1.1–12.7, *P* = 0.035), indicating that DDC can predict disease progression in the early asymptomatic stages. For illustration purposes, Extended Data Fig. 2 shows the Kaplan–Meier curve for the survival function.

**Extended Data Fig. 3:**
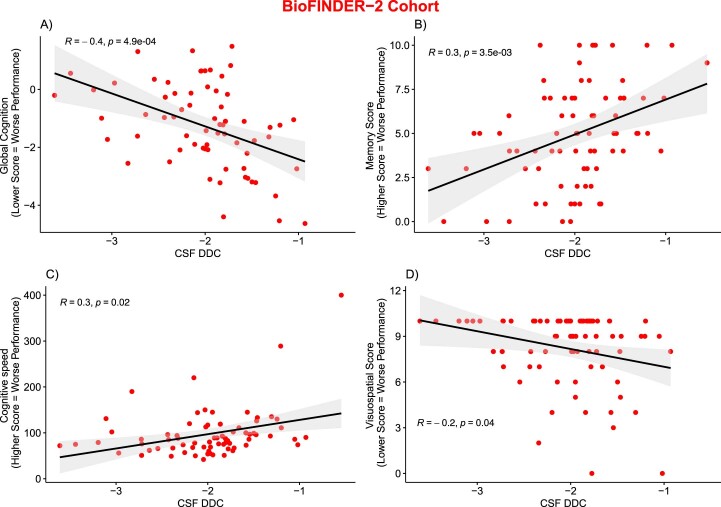
Cerebrospinal fluid DDC levels are associated with worse motor and cognitive functions in clinical LBD. Higher cerebrospinal fluid (CSF) DDC levels are associated with **a**) worse global cognition measured with the modified Preclinical Alzheimer Cognitive Composite (mPACC), **b**) higher delayed memory recall from ADAS-Cog (indicating worse performance), **c**) higher AQT form color scores, (indicating worse performance) and **d**) worse visuospatial scores measured with visual object and space perception battery – cube analysis subtest in patients with clinical LBD.

To investigate whether DDC was associated with clinical dysfunction, we assessed the relationship between this biomarker with cognitive assessments in patients with clinical LBD using partial correlations, while adjusting for the effects of age, sex and education. These analyses revealed that increasing DDC levels were associated with worse global cognition (mPACC: *ρ* (Spearman’s correlation coefficient) = −0.4, *P* = 4.9 × 10^−4^), low memory performance (delayed memory recall from the Alzheimer’s Disease Assessment Scale–Cognitive Subscale (ADAS-Cog): *ρ* = 0.3, *P* = 3.5 × 10^−3^), worse cognitive speed (AQT: *ρ* = 0.3, *P* = 0.02) and visuospatial abilities (visual object and space perception battery–cube analysis subtest: *ρ* = -0.2, *P* = 0.04), indicating that DDC has an important clinical value for assessing cognitive dysfunction (Extended Data Fig. 3a–d). There was no significant relation with motor symptoms (UPDRS III: *ρ* = 0.2, *P* = 0.1) when using data from patients with LBD. However, in a combined group of controls and patients with LBD, higher DDC levels were associated with more severe motor symptoms (*ρ* = 0.4, *P* = 7.3 × 10^−10^).

**Extended Data Fig. 4:**
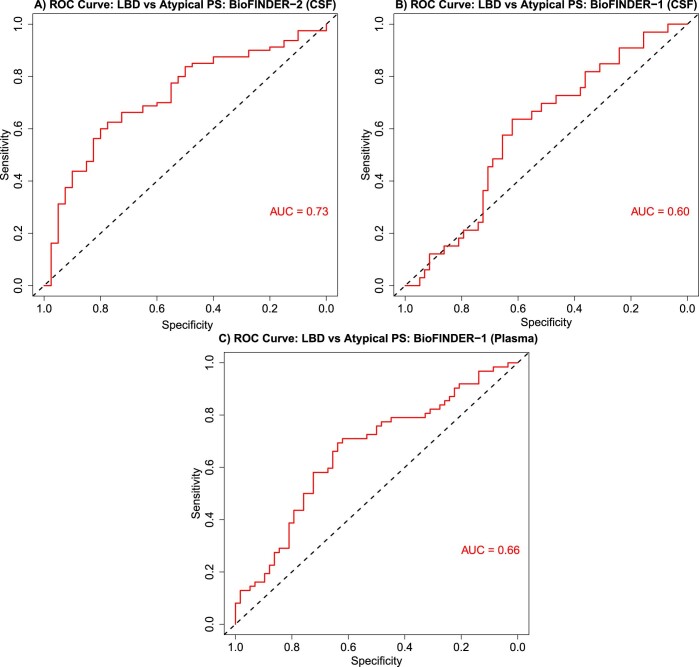
Results from the receiver operating curve analyses for the group comparisons. **a**) LBD vs Atypical PS: BioFINDER−2 (CSF), **b**) LBD vs Atypical PS: BioFINDER−1 (CSF), **c**) ROC Curve: LBD vs Atypical PS: BioFINDER−1 (Plasma). All results were obtained using an analysis of covariance (ANCOVA) while adjusting for age and sex and controlling for multiple comparisons using Bonferroni.

To establish whether our findings could also be observed in patients with atypical PS (that is, MSA, PSP, CBS) (*n* = 40), we compared their CSF DDC levels with controls (*n* = 347). We found that DDC was again significantly elevated in these atypical disorders (*F*_1,385_ = 45.2, *P*_Bonferroni-corrected_ = 3.3 × 10^−10^, AUC = 0.79, sensitivity = 0.89, specificity = 0.61) (Fig. 2e,j), suggesting that DDC might be a marker of dopaminergic dysfunction rather than α-synuclein-based Lewy body pathology. Within atypical PS, no differences were found between patients with MSA (*n* = 11) and PSP (*n* = 24) (*F*_2,38_ = 1.0, *P* = 0.32) or across the CBS (*n* = 5), MSA and PSP groups (*F*_3,37_ = 1.4, *P* = 0.26). Compared to the control group, DDC was significantly elevated in each atypical Parkinsonian subgroup (PSP: *F*_2,367_ = 8.2, *P*_Bonferroni-corrected_ = 8.7 × 10^−7^; MSA: *F*_2,354_ = 8.5, *P*_Bonferroni-corrected_ = 1.1 × 10^−6^; CBS: *F*_2,348_ = 3.3, *P*_Bonferroni-corrected_ = 3.3 × 10^–3^). As expected, in the entire group with atypical parkinsonism, there were fewer patients who were SAA^+^ (7.5%) (*n* = 3: two with PSP and one with CBS) compared to those with LBD (91.3%) (*n* = 74: 44 with PD and 30 with DLB). When we ran a binary logistic regression analysis with group (LBD versus atypical PS) as a dependent variable and both DDC level and SAA status as independent predictors, we found that SAA was a highly significant variable that discriminated LBD from atypical PS(SAA: *β* = 5, *P* = 3.6 × 10^−11^, AUC = 0.95, sensitivity = 0.93, specificity = 0.93), in contrast to DDC (*β* = 0.6, *P* = 0.01, AUC = 0.73, sensitivity = 0.37, specificity = 0.89) (Extended Data Fig. 4a). In line with this, DDC was the only variable discriminating atypical PS from controls (SAA: *β* = −0.6, *P* = 0.3; DDC: *β* = 1.2, *P* = 2 × 10^−8^), whereas both DDC and SAA discriminated clinical LBD from controls (SAA: *β* = 4.7, *P* = 6 × 10^−21^; DDC: *β* = 2.2, *P* = 1.1 × 10^−16^). These results suggest that DDC and SAA might be complementary biomarkers for the diagnosis and discrimination of LBD and atypical Parkinsonian disorders.

We performed a multinomial logistics regression as a sensitivity analysis to assess the robustness of the receiver operating characteristic (ROC) AUC statistics in the BioFINDER 2 cohort. The results showed that DDC could significantly differentiate LBD subtypes (DLB: *β* = 3.3, *P* = 3 × 10^−12^; PD: *β* = 2.6, *P* = 1.5 × 10^−13^) from controls. It also showed a good discriminative ability of DDC between atypical PD subtypes (CBS: *β* = 2.1, *P* = 7.4 × 10^−3^; MSA: *β* = 2.3, *P* = 5.1 × 10^−6^; PSP: *β* = 1.5, *P* = 5.6 × 10^−5^) and controls (Supplementary Results).

**Extended Data Fig. 5:**
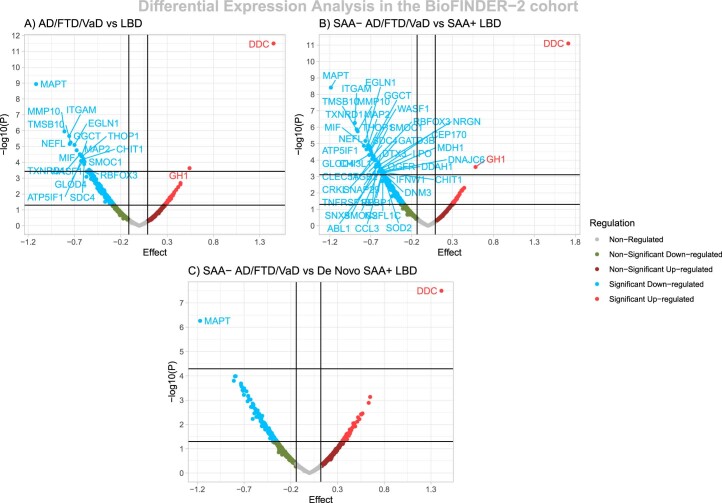
Differential expression analyses of patients with LBD compared to non-Parkinsonian disorders in BioFINDER-2. **a**) Results comparing all non-Parkinsonian disorders (Alzheimer’s disease, AD; fronto-temporal dementia, FTD; vascular dementia, VaD) to Lewy body disease (LBD) patients. **b**) Results comparing AD/FTD/VaD with a negative α-synuclein Seed Amplification Assay (SAA) (SAA-) to LBD patients with a positive SAA (SAA+). **c**) Results comparing SAA- AD/FTD/VaD patients to SAA+ *de novo* LBD patients. The upper horizontal indicates proteins that survived FDR corrections. The strongest top hit corresponded to DOPA decarboxylase (DDC, aka aromatic L-amino acid decarboxylase).

At this point, it was not yet clear whether DDC levels are affected in other neurodegenerative disorders. Thus, we ran differential expression analyses comparing patients with Parkinsonian traits with 214 individuals with AD (*n* = 172), FTD (*n* = 23) or vascular dementia (*n* = 19) from the BioFINDER 2 cohort. These analyses showed that DDC was significantly upregulated in LBD (*β* = 1.5, *P*_FDR_ = 9.4 × 10^−9^, AUC = 0.83, specificity = 0.74, specificity = 0.81), SAA^+^ LBD (*β* = 1.7, *P*_FDR_ = 2.3 × 10^−8^, AUC = 0.85, sensitivity = 0.74, specificity = 0.85) and de novo SAA^+^ LBD (*β* = 1.4, *P*_FDR_ = 9.3 × 10^−5^, AUC = 0.83, sensitivity = 0.69, specificity = 0.86) compared to non-Parkinsonian neurodegenerative disorders (AD, FTD and vascular dementia) (Extended Data Fig. 5a–c), indicating that changes in this biomarker are specific for Parkinsonian diseases (Supplementary Tables 5–7).

### Replication of the study in CSF and plasma

**Fig. 3:**
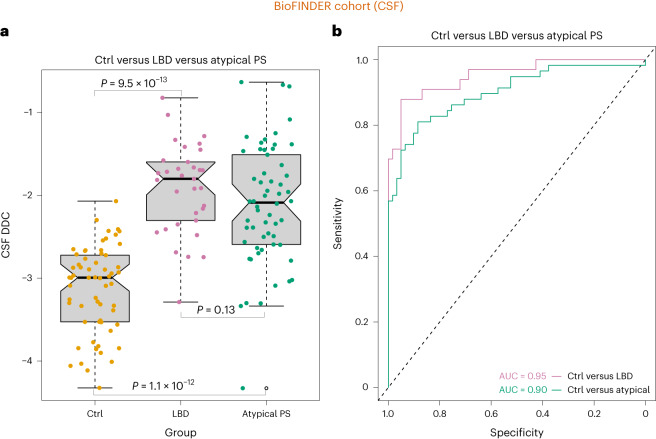
Increased CSF DDC levels in clinical LBD and atypical PS in an independent cohort. **a**, Higher CSF DDC levels were observed in all patients with LBD and atypical PS compared to CUIs or controls, *n* = 152 (patients with LBD (PD = 32, DLB = 1), 61 controls (CUIs = 29, SCD = 32) and 58 atypical Parkinsonian disorders (MSA = 30, PSP = 28). The box denotes the limits of the IQR, which is calculated by dividing the median by the range of the data; the whiskers extend beyond the box to a maximum of 1.5 times the IQR. **b**, Results from the ROC analyses for the group comparisons with the corresponding AUCs. All results were obtained using an ANCOVA while adjusting for age and sex and controlling for multiple comparisons using Bonferroni correction. All *P* values are two-sided.

**Extended Data Fig. 6:**
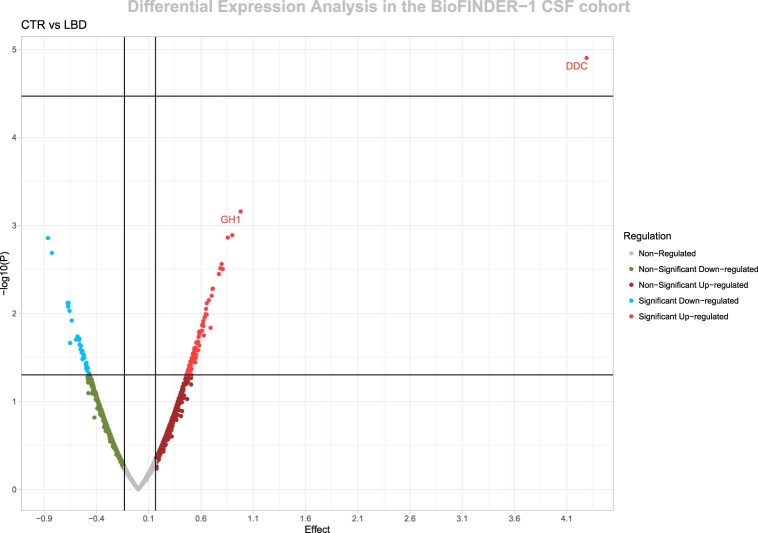
Differential expression analyses of patients with LBD compared to controls in the BioFINDER-1 cohort with CSF samples. Results comparing all clinical unimpaired individuals or controls (CTR) to Lewy body disease (LBD) patients. The upper horizontal indicates proteins that survived FDR corrections. The strongest top hit corresponded to DOPA decarboxylase (DDC, also known as aromatic L-amino acid decarboxylase).

To determine whether our findings were not due to the characteristics of a particular cohort, we ran differential expression analyses of the same 2,943 proteins using CSF samples from 33 patients with LBD (PD = 32, DLB = 1) and 61 controls (CUIs = 29, subjective cognitive decline (SCD) = 32) from an independent sample, the BioFINDER 1 cohort, which does not overlap with the BioFINDER 2 cohort used in the previous analyses (Supplementary Table 8). These analyses showed that DDC levels were again significantly upregulated in patients with LBD compared to controls, being the strongest top hit (Extended Data Fig. 6 and Supplementary Table 9). Furthermore, DDC differentiated patients with LBD from controls with high accuracy (*F*_1,92_ = 113.6, *P* = 9.5 × 10^−13^, AUC = 0.95, sensitivity = 0.86, specificity = 0.90) (Fig. 3a,b), replicating the results we obtained in BioFINDER 2 and suggesting that higher DDC in patients with LBD is generalizable to the population with LBD. In BioFINDER 1, we also evaluated a sample of atypical Parkinsonian disorders (total, *n* = 58; MSA, *n* = 30; PSP, *n* = 28), who showed higher DDC levels compared to controls (*F*_1,117_ = 77.9 *P* = 1.1 × 10^−12^, AUC = 0.90, sensitivity = 0.80, specificity = 0.90) (Fig. 3a,b), confirming that DDC is a likely marker for dopaminergic dysfunction. Similarly to BioFINDER 2, DDC was not a good biomarker to discriminate atypical Parkinsonian disorders from LBD in BioFINDER 1 (AUC = 0.60, sensitivity = 0.95, specificity = 0.05) (Extended Data Fig. 4b).

**Fig. 4:**
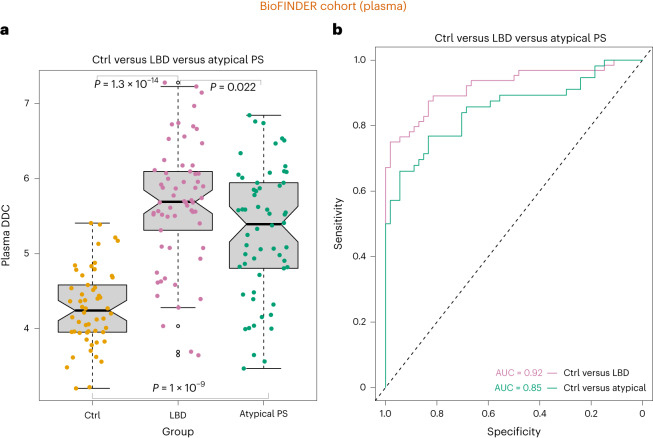
Increased plasma DDC levels in clinical LBD and atypical PS in an independent cohort. **a**, Higher plasma DDC levels were observed in all patients with LBD and atypical PS compared to CUIs or controls, *n* = 174 (64 patients with LBD; PD = 2; DLB = 2), 54 controls (CUIs = 29, showing SCD = 25) and 56 atypical PS (MSA = 30, PSP = 26). The box denotes the limits of the IQR, which is calculated by dividing the median by the range of the data; the whiskers extend beyond the box to a maximum of 1.5 times the IQR. **b**, Results from the ROC analyses for the group comparisons with the corresponding AUCs. All results were obtained using an ANCOVA while adjusting for age and sex and controlling for multiple comparisons using Bonferroni correction. All *P* values are two-sided.

**Extended Data Fig. 7:**
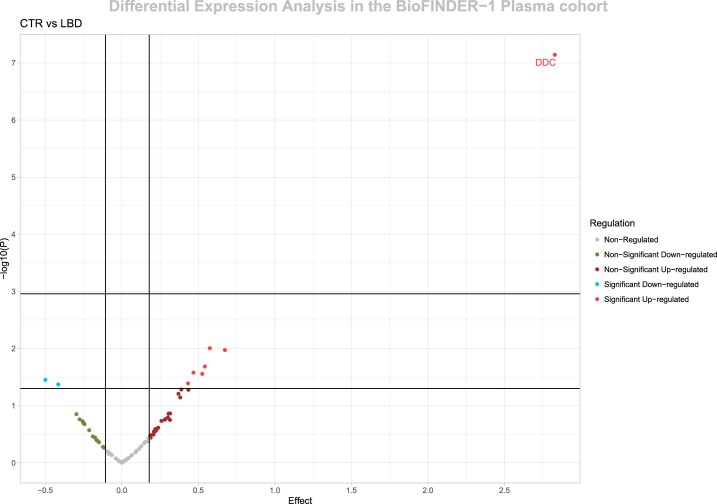
Differential expression analyses of patients with LBD compared to controls in the BioFINDER-1 cohort with plasma samples. Results comparing all clinical unimpaired individuals or controls (CTR) to Lewy body disease (LBD) patients. The upper horizontal indicates proteins that survived FDR corrections. The strongest top hit corresponded to plasma DOPA decarboxylase (DDC, also known as aromatic L-amino acid decarboxylase).

**Extended Data Fig. 8:**
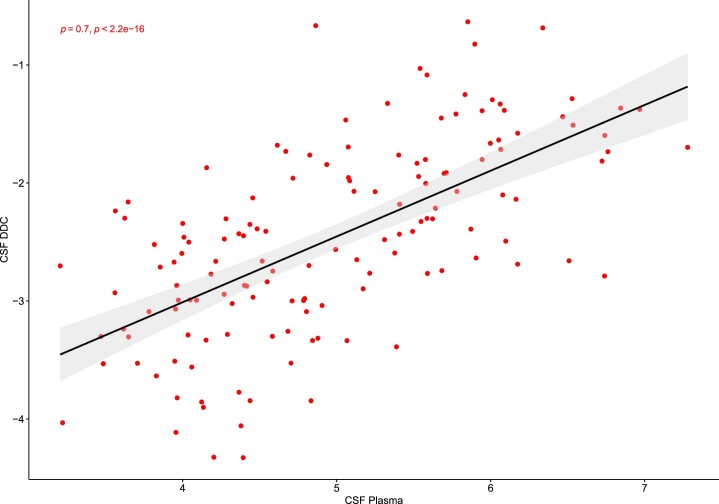
Relationship between CSF and plasma DDC in the BioFINDER-1 cohort. The plot shows a significant relationship between the levels of DDC measures in the cerebrospinal fluid (CSF) and plasma.

Because plasma biomarkers could be more easily implemented in clinical practice and drug trials, we quantified plasma concentrations of 92 proteins (including DDC) using the Olink platform^12^ in 174 individuals (64 patients with LBD; PD, *n* = 2; DLB, *n* = 2), 54 controls (CUIs, *n* = 29; showing SCD, *n* = 25) and 56 atypical PS (MSA, *n* = 30; PSP, *n* = 26) from the BioFINDER 1 cohort (Supplementary Table 10). Like our CSF findings, plasma DDC levels were significantly higher in individuals with LBD compared to controls (Extended Data Fig. 7 and Supplementary Table 11). Moreover, plasma DDC showed high accuracy for discriminating controls from individuals with LBD (*F*_1,116_ = 111.6, *P* = 1.3 × 10^−14^, AUC = 0.92, sensitivity = 0.98, specificity = 0.82) (Fig. 4a,b) as well as atypical Parkinsonian disorders (MSA, PSP) (*F*_1,110_ = 56.5 *P* = 1 × 10^−9^, AUC = 0.85, sensitivity = 0.89, specificity = 0.74) (Fig. 4a,b). When comparing LBD with atypical Parkinsonian disorders, the results were similar to our previous results showing that DDC was unable to accurately discriminate LBD from atypical Parkinsonian disorders (AUC = 0.66, sensitivity = 0.52, specificity = 0.73) (Extended Data Fig. 4c). Finally, we found high correlations between CSF and plasma DDC levels (Spearman *ρ* = 0.7, *P* < 2.2 × 10^−16^) in a subsample of 142 BioFINDER 1 participants (LBD, *n* = 33; atypical PS, *n* = 56; controls, *n* = 53; of which 29 were CUIs and 24 had SCD), in whom both measures were available (Extended Data Fig. 8).

## Discussion

Collectively, our findings show that DDC is a unique and very promising biomarker for LBD and atypical Parkinsonian disorders. Mutations in the *DDC* gene result in a severe deficiency of dopamine, serotonin, norepinephrine and epinephrine, implying that this gene has an important role in the production of neurotransmitters in the brain^15^. Furthermore, DDC is essential to the formation of dopamine from exogenous l‐DOPA; treatment with l‐DOPA is normally combined with an inhibitor of DDC (carbidopa), which does not enter the central nervous system and thereby reduces l-DOPA-associated production of dopamine outside the brain. Therefore, it was important that DDC levels were upregulated also in drug-naive (de novo) patients with Parkinsonian symptoms and in nontreated individuals with preclinical LBD in our study.

It could be hypothesized that the increased production of DDC observed in the present study is a means for neurons that normally receive dopaminergic input (such as the neurons in the striatum) to compensate for low dopaminergic levels. In fact, in a previous rat model, DDC activity was upregulated soon after lesions in dopaminergic neurons, suggesting the involvement of compensatory mechanisms^16^. Consequently, increased levels of DDC could be a marker of reduced dopamine signaling in the brain, a key feature of both LBD and atypical Parkinsonian disorders. Another potential explanation for increased CSF DDC levels could be the increases in peripheral DDC in Parkinsonian disorders with leakage of peripheral DDC into the CSF; future studies are needed to explore this further.

Since α-synuclein SAA is an accurate biomarker for LBD but not always for atypical Parkinsonian disorders (which lack Lewy bodies), we suggest that DDC and α-synuclein SAA may be combined in clinical practice in the future, where high DDC and abnormal α-synuclein SAA would indicate LBD, whereas high DDC and normal α-synuclein SAA would indicate an atypical Parkinsonian disorder. A similar distinction of LBD from atypical PS has already been proposed using SAA in combination with neurofilament light chain^17–21^; however, the latter, in contrast to DDC, is a rather unspecific neurodegeneration biomarker that is affected across several neurodegenerative disorders, such as amyotrophic lateral sclerosis and FTD, among many others^22^. Of note, the real-time quaking-induced conversion (RT-QuIC) assay we used in the current study does not detect α-synuclein pathology in the α-synucleinopathy MSA^23^ (and is also negative for cases with 4R tauopathies like PSP and CBS) compared to other assays such as protein misfolding cyclic amplification)^24^, which detects α-synuclein aggregation in patients with MSA because it seems to be sensitive to structural differences in the α-synuclein aggregates between MSA and PD^25,26^. Thus, the combination of DDC with the specific RT-QuIC assay we used could be applied to discriminate LBD from atypical Parkinsonian disorders, which sometimes are difficult to distinguish due to overlapping symptoms. Moreover, it is often difficult for dementia disorder experts to distinguish between patients with DLB and patients with other dementia disorders like AD and vascular dementia. Dopamine transporter imaging is usually used for this purpose, but this method includes radiation, is expensive and not widely available. Further, early stages of Parkinsonian disorders, particularly in older adults, can be challenging to differentiate from other non-Parkinsonian conditions that cause slow movements and poor balance. Finally, the biomarker we found also holds great promise to identify preclinical stages of Parkinsonian disorders, which will be very important for the detection of individuals in the early disease stages for trials evaluating innovative disease-modifying therapies. In particular, we found that DDC predicted conversion from preclinical to clinical LBD, indicating that it has an important prognostic value. Finally, two limitations related to our work should be recognized. First, in addition to Olink, we did not investigate any other technique that can reliably measure DDC protein levels in the CSF or plasma. Future studies should study in detail which antibody pairs best detect the DDC variant that can optimally distinguish Parkinsonian disorders from other conditions. Second, due to sample size, our survival analysis assessing whether DDC can predict conversion to LBD in CUIs only included 12 converters over a period of approximately 1–4 years. Thus, additional longitudinal studies with a larger follow-up, number of visits and number of individuals who convert to LBD are needed to verify our conclusion that DDC can predict disease progression in the early asymptomatic stages.

In summary, our findings suggest that CSF DDC is a highly promising biomarker for Parkinsonian disorders. In particular, if the plasma DDC results are replicated in other cohorts, this biomarker could be important for early and even preclinical detection of Parkinsonian disorders and predict future conversion to clinical LBD. Moreover, when combined with the specific α-synuclein SAA used in our study, DDC might improve the diagnostic workup of Parkinsonian disorders in clinical practice. Although we replicated our findings in an independent cohort, future studies are needed to analyze DDC in several cohorts and assess the generalizability of our results across multiple samples. Furthermore, we did not have longitudinal DDC data, which will be crucial to determine the clinical value of changes in this biomarker over time.

## Methods

### Participants

We included 682 individuals from the prospective Swedish BioFINDER 2 cohort (ClinicalTrials.gov registration: NCT03174938)^27^, which has the aim of identifying and developing biomarkers for the diagnosis of neurodegenerative diseases. All participants were recruited at the Skåne University hospital, Sweden between 2017 and 2020 and included 347 controls, 81 patients with LBD, 40 patients with atypical PS and 214 patients with other non-Parkinsonian neurodegenerative disorders, who all underwent lumbar puncture and clinical examinations. The group with LBD consisted of 48 patients with PD and 33 with DLB (of which 50 were drug-naive or de novo). The atypical Parkinsonian group included 24 patients with PSP, 11 with MSA and five with CBS (of which 21 were drug-naive or de novo). Finally, the group with other neurodegenerative disorders consisted of 172 patients with AD, 23 with FTD and 19 with vascular dementia. The UPDRS part III^28^, was used to assess motor function (Supplementary Tables 1, 8 and 9). The levodopa-equivalent daily dose was calculated, when available, in patients with Parkinsonian disorder^29^. Global cognition was assessed using the mPACC^30^ containing tests of memory, executive, attention and verbal functions (lower score, worse global cognition). We also assessed more specific cognitive domains including cognitive speed (AQT; higher score, worse cognitive speed)^31,32^, memory function (delayed memory recall from the ADAS-Cog; higher score, worse memory)^33^ and visuospatial function (visual object and space perception battery, cube analysis subtest; lower score, worse visuospatial function)^34^ (Supplementary Table 1).

To replicate our findings in an independent cohort, we included 152 individuals from the Swedish BioFINDER 1 study (ClinicalTrials.gov registration: NCT01208675)^35^ recruited between 2007 and 2015. In this cohort, CSF samples were analyzed in 61 CUIs (controls), 33 individuals with LBD (31 with PD, one with PD dementia and one with DLB) and 58 individuals with atypical PS (28 with PSP and 30 with MSA). For the plasma analysis in the same cohort, we included 174 individuals, of whom 54 were clinically unimpaired (controls), 64 were diagnosed with LBD (36 with PD, 26 with PD dementia and two with DLB) and 56 were diagnosed with an atypical PS (26 with PSP and 30 with MSA).

In both cohorts, CUIs or controls consisted of CUIs and participants with SCD who performed within normal ranges on a large cognitive test battery applied by experienced neuropsychologists. Briefly, they were required to (1) be 40–100 years old, (2) have a Mini-Mental State Examination score equal to or greater than 24 points and (3) be fluent in Swedish. Importantly, none of the included control participants fulfilled the clinical criteria for PD, prodromal DLB, atypical parkinsonism or any other neurological disorder at baseline. Patients with LBD were required to (1) fulfill the criteria for PD^36^, dementia due to DLB^37^ or PD dementia^38^ and (2) be fluent in Swedish. Patients with atypical Parkinsonian disorders were required to (1) meet the consensus statement for MSA^39^, the criteria according to the report of the National Institute of Neurological Disorders and Stroke-Society for PSP International Workshop^40^ or the diagnosis guidelines for CBS^41^, and (2) be fluent in Swedish. Patients with other non-Parkinsonian neurodegenerative disorders were required to (1) fulfill the Diagnostic and Statistical Manual of Mental Disorders, Fifth Edition criteria for dementia (major neurocognitive disorder) due to AD^42^, FTD^43^ or vascular dementia^43^, and (2) be fluent in Swedish. Exclusion criteria for all groups were (1) having serious unstable systemic illness that made it difficult to participate in the study, (2) current critical alcohol or substance misuse and (3) refusing lumbar puncture.

All participants gave written informed consent before entering the study. The study procedure was approved by the local ethics committee at Lund University, Sweden (ethical approval case no. 2016/1053 for BioFINDER 2, case no. 2010/156 for BioFINDER 1) and conducted according to the Declaration of Helsinki (Seventh revision, 2013).

A detailed description of the number of individuals included in each group for each cohort is provided in the Supplementary Methods.

### Lumbar puncture

Lumbar puncture was performed in the L3/L4 or L4/L5 interspace while individuals were not fasting^11^. The CSF samples were collected in polypropylene tubes, gently mixed to avoid gradient effects, centrifuged within 30 min at +4 °C at 2,000*g* for 10 min to remove cells and debris, and then stored in aliquots at −80 °C pending biochemical analysis.

### CSF and plasma analyses

All samples were analyzed using the Olink Explore 3072 platform, developed by Olink Proteomics^13^. The measurements of 2,943 proteins for the CSF samples of BioFINDER 2 and BioFINDER 1 in addition to the 92 proteins for the plasma samples of BioFINDER 1 were performed using technology based on a proximity extension assay, in accordance with the protocol of the manufacturer. First, antigens were incubated with pairs of antibodies that included DNA oligonucleotides bound to each of the proteins we wanted to measure. Oligonucleotides in close proximity were used to create a template for hybridization and extension, and PCR was used for preamplification. Specific primers were digested on a real-time quantitative PCR chip after digestion of residual primers and using a Biomark HD Instrument. Proteins were quantified as a normalized protein expression log_2_ scale.

To identify the presence of underlying α‐synuclein aggregation in the BioFINDER 2 participants, an RT-QuIC seed amplification assay using K23Q recombinant α‐synuclein was applied to the CSF samples^11^. Briefly, as previously described, reactions were performed in black 96‐well plates that were preloaded with six glass beads; quadruplicate reactions were seeded with 15 μl CSF. Each RT‐QuIC reaction mix was 85 μl of solution with final reaction concentrations of 40 mM sodium phosphate buffer, 170 mM NaCl, 0.1 mg ml^−1^ K23Q recombinant α-synuclein, 10 μM thioflavin T and 0.0015% SDS. The plates were closed with a plate sealer film and incubated at 42 °C in a BMG FLUOstar Omega plate reader for at least 48 h and subjected to cycles of 1 min shaking and 1 min rest for at least 48 h. Thioflavin T fluorescence measurements were taken every 45 min with fluorimeter gain settings adjusted to maintain fluorescence responses within an unsaturated range. The fluorescence threshold was calculated individually for each 96‐well plate to account for differences between plate readers. Positive reactions were those exceeding 10% of the maximum value obtained on the same plate from any individual positive reaction. All samples were subjected to a first round of blinded RT-QuIC testing in quadruplicate: samples with zero positive reactions within 48 h were deemed negative; samples giving three or four positive wells were considered positive.

### Statistics and reproducibility

All statistical analyses were performed in R (v.4.2.2). To identify unique proteins that can detect LBD, we first conducted differential expression analysis on the rank inverse-normalized protein levels from the Olink platform^12^ using a generalized linear model with the logit link function and binomial distribution to identify significantly upregulated or downregulated proteins in different groups (all LBD, SAA^+^ LBD, de novo SAA^+^ LBD, atypical PS compared to controls or other non-Parkinsonian neurodegenerative disorders) in the BioFINDER 2 cohort, while adjusting for age and sex and correcting for multiple comparisons with FDR. To assess the relationship between DDC levels with motor function, global cognition, cognitive speed, memory and visuospatial abilities, partial correlation analyses were then performed, while controlling for age, sex and education.

Afterwards, to assess the utility of DDC in preclinical LBD and atypical PS, an ANCOVA was applied using DDC as the dependent variable, and LBD or controls, SAA^+^ or SAA^−^ controls (BioFINDER 2), atypical PS or controls (BioFINDER 2) or LBD or non-Parkinsonian neurodegenerative disorders as a factor, while adjusting for covariates. In addition, we conducted an ANCOVA using DDC as the dependent variable and the subgroups of atypical PS, LBD and controls (BioFINDER 2) as a factor to compare the various subgroups.

We also ran a Cox regression model to test if DDC could predict progression to clinical LBD in cognitively normal or preclinical SAA^+^ cases, while adjusting for age and sex.

To test whether elevated DDC levels could also be observed in other cohorts, we ran again differential expression analysis on protein levels from the Olink platform^13^ in the BioFINDER 1 cohort (all LBD, de novo LBD and atypical PS compared to controls).

To examine the ability of substantial proteins to discriminate different groups, we ran ROC curve analyses with 5,000 bootstrapped samples and calculated the AUC, accuracy, specificity and confidence bands for all group comparisons.

Finally, to assess the relationship between CSF and plasma DDC levels in the BioFINDER 1 cohort, we ran Spearman correlation analyses.

No statistical methods were used to predetermine sample sizes but our sample sizes are similar to those reported in previous publications^44,45^. Data collection and analysis were not randomized nor performed blind to the experimental groups.

### Reporting summary

Further information on research design is available in the Nature Portfolio Reporting Summary linked to this article.

### Supplementary information

Supplementary Information Supplementary Methods and Results.

Reporting Summary

Supplementary Tables Supplementary Tables 1–13.

## Data Availability

Anonymized data will be shared upon request from a qualified academic investigator for the sole purpose of replicating the procedures and results presented in the article and providing that the data transfer is in agreement with European Union legislation on the general data protection regulation and decisions by the ethical review board of Sweden and Region Skåne, which should be regulated in a material transfer agreement. The corresponding author can be contacted for data access. A response to the request shall be given within 2 weeks.
