# Supplementary material for: DOPA decarboxylase is an emerging biomarker for Parkinsonian disorders including preclinical Lewy body disease
Source: Nat Aging. 2023 Sep 18;3(10):1201–9. doi: 10.1038/s43587-023-00478-y (PMC10570139; doi:10.1038/s43587-023-00478-y)
Supplement: Supplementary file 1 — Supplementary Methods and Results. [file 43587_2023_478_MOESM1_ESM.pdf]

nature aging

Letter

https://doi.org/10.1038/s43587-023-00478-y

DOPA decarboxylase is an emerging biomarker for Parkinsonian disorders including preclinical Lewy body disease

In the format provided by the authors and unedited

Supplementary Materials

Table of content

Supplementary Methods

- Cohort Description
  - BioFINDER-2
  - BioFINDER-1
- Multinomial Logistic regression

Results

- Multinomial Logistic regression

Supplementary Methods

Cohort Description

BioFINDER-2

In this cohort, we had 982 subjects with CSF samples, which were analysed to determine the concentrations of 2943 proteins.

Out of these 982 cases, a total of 446 did not have a diagnosis associated with a specific neurodegenerative disease: a) 254 subjects were cognitively unimpaired (CU), b) 93 had subjective cognitive decline (SCD) and c) 99 had mild cognitive impairment (MCI). A control group was formed by combining a) and b) (254 CU + 93 SCD; total = 347 controls), whereas the 93 MCI subjects were excluded. In addition, there were 82 subjects with a PD (n = 49) or DLB (n = 33) diagnosis. One case among these 82 subjects had concomitant atypical parkinsonism and was therefore excluded from the LBD group (total = 81 LBD subjects). Finally, some cases were diagnosed with PSP (n = 24), MSA (n = 11) or CBS (n = 5), being therefore included in the atypical Parkinsonian syndromes (APS) group (total = 40 APS subjects).

To perform comparisons with non-parkinsonian neurodegenerative disorders, we included 172 patients with Alzheimer's disease (AD), 23 with Frontotemporal Dementia (FTD) and 19 with vascular dementia (total = 214 non-parkinsonian subjects). In addition, there were 147 samples with non-PD etiology but non-demented cognitive status. These samples were not considered to be a part of the non-PD group.

The remaining 53 samples either have brain-related or other neurodegenerative disorders and were excluded from the analysis.

BioFINDER-1

BioFINDER-1 consists of 158 subjects with CSF samples for 2943 proteins. Out of 158 subjects, 29 were CU and 38 were SCD (29 CUI + 38 SCD; total = 61 controls). In addition, 6 SCD samples with co-pathology were excluded from the control group.

In addition, 31 subjects were diagnosed with PD, 1 with PDD and 1 with DLB (total = 33 LBD subjects). Finally, 28 subjects were diagnosed with PSP and 30 with MSA (total = 58 APS subjects).

We also quantified the plasma concentrations of 92 proteins in 174 cases of BioFINDER-1. Among these, 29 were CU and 25 were SCD (29 CUI + 25 SCD; total = 54 controls); 36

were diagnosed with PD, 26 with PDD and 2 with DLB (total = 64 LBD subjects); 26 were diagnosed with PSP and 30 with MSA (total = 56 APS subjects).

Multinomial Logistic regression

We conducted a multinomial logistics regression analysis as a sensitivity analysis for assessing the robustness of the ROC-AUC statistics in the BioFinder-2 cohort. Using this model, we estimated the likelihood of discriminating PD and DLB individuals (LBD subtype) from controls (the reference category). The same analysis was performed for typical PD subtypes (CBS, MSA, and PSP). Age and gender were used as the covariate. The analysis was performed using the nnet package from R (version 4.2.2).

Results

Multinomial Logistic regression

The multinomial model using the control as a reference category showed that DDC could significantly differentiate DLB from controls (β=3.3, p=3e-12) and PD from controls (β=2.6, p=1.5e-13) (Supplementary Table 12).

Analyzing the atypical PD subtypes also showed that DDC was able to significantly differentiate CBS (β=2.1, p=7.4e-3), MSA (β=2.3, p=5.1e-6) and PSP (β=1.5, p=5.6e-5) from controls (Supplementary table 13).
