# Supplementary material for: DOPA decarboxylase is an emerging biomarker for Parkinsonian disorders including preclinical Lewy body disease
Source: Nat Aging. 2023 Sep 18;3(10):1201–9. doi: 10.1038/s43587-023-00478-y (PMC10570139; doi:10.1038/s43587-023-00478-y)
Supplement: Supplementary file 2 — Reporting Summary [file 43587_2023_478_MOESM2_ESM.pdf]

nature portfolio

Corresponding author(s): Oskar Hansson

Last updated by author(s): Jul 24, 2023

Reporting Summary

Nature Portfolio wishes to improve the reproducibility of the work that we publish. This form provides structure for consistency and transparency in reporting. For further information on Nature Portfolio policies, see our Editorial Policies and the Editorial Policy Checklist.

Statistics

For all statistical analyses, confirm that the following items are present in the figure legend, table legend, main text, or Methods section.

n/a	Confirmed	
☐	☒	The exact sample size (*n*) for each experimental group/condition, given as a discrete number and unit of measurement
☐	☒	A statement on whether measurements were taken from distinct samples or whether the same sample was measured repeatedly
☐	☒	The statistical test(s) used AND whether they are one- or two-sided<br>*Only common tests should be described solely by name; describe more complex techniques in the Methods section.*
☐	☒	A description of all covariates tested
☐	☒	A description of any assumptions or corrections, such as tests of normality and adjustment for multiple comparisons
☐	☒	A full description of the statistical parameters including central tendency (e.g. means) or other basic estimates (e.g. regression coefficient) AND variation (e.g. standard deviation) or associated estimates of uncertainty (e.g. confidence intervals)
☐	☒	For null hypothesis testing, the test statistic (e.g. F, t, r) with confidence intervals, effect sizes, degrees of freedom and P value noted<br>*Give P values as exact values whenever suitable.*
☒	☐	For Bayesian analysis, information on the choice of priors and Markov chain Monte Carlo settings
☒	☐	For hierarchical and complex designs, identification of the appropriate level for tests and full reporting of outcomes
☒	☐	Estimates of effect sizes (e.g. Cohen's *d*, Pearson's *r*), indicating how they were calculated

Our web collection on statistics for biologists contains articles on many of the points above.

Software and code

Policy information about availability of computer code

Data collection	No software was used.
Data analysis	R (version 4.2.2) was used for statistical analyses as described in the manuscript. survival package 3.5-5

For manuscripts utilizing custom algorithms or software that are central to the research but not yet described in published literature, software must be made available to editors and reviewers. We strongly encourage code deposition in a community repository (e.g. GitHub). See the Nature Portfolio guidelines for submitting code & software for further information.

Data

Policy information about availability of data

All manuscripts must include a data availability statement. This statement should provide the following information, where applicable:

- Accession codes, unique identifiers, or web links for publicly available datasets
- A description of any restrictions on data availability
- For clinical datasets or third party data, please ensure that the statement adheres to our policy

Pseudonymized data will be shared by request from a qualified academic investigator for the sole purpose of replicating procedures and results presented in the article and as long as data transfer is in agreement with EU legislation on the general data protection regulation and decisions by the Swedish Ethical Review Authority and Region Skåne, which should be regulated in a material transfer agreement. Corresponding author maybe contacted for data access. A response to the request shall be given in two weeks of time.

Research involving human participants, their data, or biological material

Policy information about studies with human participants or human data. See also policy information about sex, gender (identity/presentation), and sexual orientation and race, ethnicity and racism.

Reporting on sex and gender	There is almost 1:1 male female ratio for the cohort. Sex is reported in the the descriptive tables (1, 8 and 10) and is a covariate in the statical analyses.
Reporting on race, ethnicity, or other socially relevant groupings	Please see the supplementary tables (1, 8 and 10) of the manuscript. Details about confounding variable is provided in the manuscript
Population characteristics	Please see the supplementary tables (1, 8 and 10) of the manuscript.
Recruitment	Recruitment is described in the manuscript, and on www.clinicaltrials.gov for NCT03174938 (BioFINDER-2) and NCT01208675 (BioFINDER-1). All participants were recruited at the Skåne University hospital, Sweden between 2017 and 2020 and included 347 controls, 81 patients with LBD, 40 patients with atypical Parkinsonian syndromes and 214 patients with other non-Parkinsonian neurodegenerative disorders, who all underwent lumbar puncture and clinical examinations. There was no selection bias.
Ethics oversight	The study procedure was approved by the local ethics committee at Lund University in Sweden and conducted according to the Helsinki Declaration.

Note that full information on the approval of the study protocol must also be provided in the manuscript.

Field-specific reporting

Please select the one below that is the best fit for your research. If you are not sure, read the appropriate sections before making your selection.

☒ Life sciences ☐ Behavioural & social sciences ☐ Ecological, evolutionary & environmental sciences

For a reference copy of the document with all sections, see nature.com/documents/nr-reporting-summary-flat.pdf

Life sciences study design

All studies must disclose on these points even when the disclosure is negative.

Sample size	All study participants with available Olink data were included.
Data exclusions	Exclusion and inclusion criteria is provided in the supplementary note method section.
Replication	As described in the manuscript, 2 independent cohorts were used and the findings were successfully replicated in both the cohorts
Randomization	NA, as this is an observational study and not an interventional study
Blinding	All samples were analysed at Olink in a blinded fashion.

Reporting for specific materials, systems and methods

We require information from authors about some types of materials, experimental systems and methods used in many studies. Here, indicate whether each material, system or method listed is relevant to your study. If you are not sure if a list item applies to your research, read the appropriate section before selecting a response.

Materials & experimental systems

n/a	Involved in the study	
☒	☐	Antibodies
☒	☐	Eukaryotic cell lines
☒	☐	Palaeontology and archaeology
☒	☐	Animals and other organisms
☐	☒	Clinical data
☒	☐	Dual use research of concern
☒	☐	Plants

Methods

n/a	Involved in the study	
☒	☐	ChIP-seq
☒	☐	Flow cytometry
☒	☐	MRI-based neuroimaging

Clinical data

Policy information about clinical studies

All manuscripts should comply with the ICMJE guidelines for publication of clinical research and a completed CONSORT checklist must be included with all submissions.

Clinical trial registration	Both studies were registered at www.clinicaltrials.gov for NCT03174938 (BioFINDER-2) and NCT01208675 (BioFINDER-1).
Study protocol	Please see www.clinicaltrials.gov for the outlines of NCT03174938 (BioFINDER-2) and NCT01208675 (BioFINDER-1).
Data collection	As described in the manuscript, in the Swedish BioFINDER-2 cohort (NCT03174938) all participants were recruited at the Skåne University hospital, Sweden between 2017 and 2020; in the Swedish BioFINDER-1 study (NCT01208675) study participants were recruited between 2007 and 2015.
Outcomes	The primary aim was to identify unique biomarkers that can detect clinical LBD and atypical Parkinsonian disorders. The secondary aim was to find biomarkers that could detect preclinical LBD (clinically unimpaired individuals with a positive SAA assay) reflecting underlying abnormal α-synuclein aggregation. our findings showed that DDC is a unique and very promising biomarker for LBD, atypical parkinsonian disorders as well as for pre-clinical LBD
